# Interactions between the *adducin 2 *gene and antihypertensive drug therapies in determining blood pressure in people with hypertension

**DOI:** 10.1186/1471-2350-8-61

**Published:** 2007-09-13

**Authors:** Sharon LR Kardia, Yan V Sun, Sara C Hamon, Ruth Ann Barkley, Eric Boerwinkle, Stephen T Turner

**Affiliations:** 1 Department of Epidemiology, University of Michigan, Ann Arbor, MI, USA; 2 Department of Human Genetics, University of Michigan, Ann Arbor, MI, USA; 3 Human Genetics Center, University of Texas Health Sciences Center, Houston, TX, USA; 4 Department of Internal Medicine and Division of Hypertension, Mayo Clinic, Rochester, MN, USA

## Abstract

**Background:**

As part of the NHLBI Family Blood Pressure Program, the Genetic Epidemiology Network of Arteriopathy (GENOA) recruited 575 sibships (n = 1583 individuals) from Rochester, MN who had at least two hypertensive siblings diagnosed before age 60. Linkage analysis identified a region on chromosome 2 that was investigated using 70 single nucleotide polymorphisms (SNPs) typed in 7 positional candidate genes, including adducin 2 (*ADD2*).

**Method:**

To investigate whether blood pressure (BP) levels in these hypertensives (n = 1133) were influenced by gene-by-drug interactions, we used cross-validation statistical methods (i.e., estimating a model for predicting BP levels in one subgroup and testing it in a different subgroup). These methods greatly reduced the chance of false positive findings.

**Results:**

Eight SNPs in *ADD2 *were significantly associated with systolic BP in untreated hypertensives (p-value < 0.05). Moreover, we also identified SNPs associated with gene-by-drug interactions on systolic BP in drug-treated hypertensives. The TT genotype at SNP rs1541582 was associated with an average systolic BP of 133 mmHg in the beta-blocker subgroup and 148 mmHg in the diuretic subgroup after adjusting for overall mean differences among drug classes.

**Conclusion:**

Our findings suggest that hypertension candidate gene variation may influence BP responses to specific antihypertensive drug therapies and measurement of genetic variation may assist in identifying subgroups of hypertensive patients who will benefit most from particular antihypertensive drug therapies.

## Background

Hypertension affects more than 50 million Americans and is the most common disease for which adults seek medical attention [1,2]. Despite the large variety and efficacy of antihypertensive drugs, less than 50% of treated hypertensives have their BP adequately controlled. It is well known that hypertensives respond heterogeneously to antihypertensive therapies, often requiring multiple medications to lower their BP. This heterogeneity reflects the multitude of factors that influence interindividual variation in the pharmacokinetic properties of drug (i.e. mechanisms of drug absorption, distribution, metabolism, or excretion) and pharmacodynamic properties (i.e. biochemical and physiological mechanisms associated with the drug target). Identifying genetic variations that influence these biochemical, physiological, and anatomical mechanisms regulating BP response to antihypertensive therapies could have a major public health impact on reducing the target organ damage due to hypertension by targeting drug interventions based on an individual's biological profile.

As part of the National Heart Lung and Blood Institute's Family Blood Pressure Program (FBPP), an extensive genetic and epidemiological research effort has been established to identify genes for hypertension and its clinical complications. As part of FBPP, positional candidate gene study of a significant linkage region on chromosome 2 (40 cM – 140 cM) implicated several hypertension susceptibility genes in this region [3]. One positional candidate gene implicated by the analysis was the *adducin 2 *gene (*ADD2*). *ADD2 *encodes the beta subunit of the cytoskeleton adducin heterodimeric protein and spans 108 kb including seventeen exons that are alternatively spliced to code for at least five known protein isoforms [4]. The adducin subunits have two structurally distinct domains: an N-terminal globular head and a C-terminal that contains phosphorylation sites and a MARCKS related domain [5]. A variant of the *Add2 *gene was also found to be associated with BP in rat [6]. Adducin has been proposed to regulate renal tubular transport of Na^+ ^reabsorption, which in turn regulates body sodium, fluid volumes and the development of hypertension [6,7].

Genes that influence risk of developing hypertension are prime candidates for influencing an individual's pharmacodynamic response to blood pressure-lowering therapies. Therefore, we examined whether single nucleotide polymorphisms (SNPs) in the *ADD2 *gene influence BP in hypertensives stratified by antihypertensive drug therapy category by testing for evidence of gene-by-drug interactions in a population-based sample of non-Hispanic white hypertensives from Rochester, MN participating in the Genetic Epidemiology Network of Arteriopathy (GENOA) study of FBPP.

One of the basic issues in genetic association studies has been the lack of replication of effects across studies. While testing replication in different populations is an important step, it does not address an emerging awareness that there are many gene-environment and gene-gene interactions underlying common traits like blood pressure [8-10]. In fact, we would not expect the replication of these types of effects unless the sample was from the same population [11]. Cross-validation is a procedure to test the sample predictive validity on an independent dataset which is often obtained by randomly splitting the whole dataset into subsets [12,13]. Cross-validation has been a critical component in gene expression microarray analysis to assess the model predictive ability [14]. In the current study, we have tried to derive a strategy that combines both replication and cross-validation to identify important genetic variations. Basically, within our sample of hypertensives, there are 8 subgroups defined by their antihypertensive therapeutic regimes. Identifying SNPs with similar effects in multiple subgroups is one form of replication. In addition, for SNPs that influence response to antihypertentive drugs we have the added evidence for cross-validation methods that they predict BP levels in independent test cases. Combined, replication and cross-validation, provided a level of confidence that simply goes to another epidemiological or clinical sample may not be appropriate.

## Methods

### Study sample

The study was approved by the Institutional Review Boards of all participating institutions (University of Texas, Mayo Clinic and University of Michigan) to be compliance with the Helsinki Declaration. Written informed consent was obtained from each participant.

Descriptions of sample ascertainment, exclusion, and phenotype collection for the FBPP have been previously described [15] and are publicly available [16]. In this study, we focused our analyses on the 1133 non-Hispanic white, hypertensive participants from the Rochester, MN field center which are part of the GENOA (Genetic Epidemiology Network of Arteriopathy) component of the FBPP. In Rochester, MN, the Mayo Clinic diagnostic index and medical record linkage system of the Rochester Epidemiology Project were used to identify all Olmsted County residents less than 60 years old who had the diagnosis of essential hypertension and received care in the county during the previous three years [17]. Eligible probands were contacted and asked whether they had any siblings living in the area. If so, the siblings were contacted, and if at least one reported the previous diagnosis of hypertension, all available members of the sibship were invited into the study.

Study visits were conducted in the morning after an overnight fast of at least eight hours. Using random zero sphygmomanometers and cuffs appropriate for arm size, three readings of blood pressure were taken in the right arm after the participant rested in the sitting position for at least five minutes; the last two readings were averaged for the analyses. Participants included in the present analyses met at least one of the following two criteria for the diagnosis of "definite" hypertension: prior diagnosis of hypertension by a physician and use of prescription antihypertensive medication reported at the study visit; *or *the second and third BP measurements obtained at the study visit averaged ≥ 140 mm Hg for systolic pressure or ≥ 90 mm Hg for diastolic pressure.

Each prescription antihypertensive drug recorded at the study visit was assigned a code number corresponding to the first six digits of the Medi-Span Generic Product Identifier [18]. This number, which identifies pharmacologically equivalent drug classes, was used to categorize agents with similar mechanisms of antihypertensive action. Participants on monotherapies were classified as taking beta-blockers, calcium-channel blockers, RAAS inhibitors, diuretics, or "other" antihypertensive drugs. Those on combination therapies were classified as taking "beta-blocker + diuretic", "beta-blocker + other antihypertensive", "diuretic + other antihypertensive" or "neither beta-blocker nor diuretic".

### Genotyping

**Figure 1:**
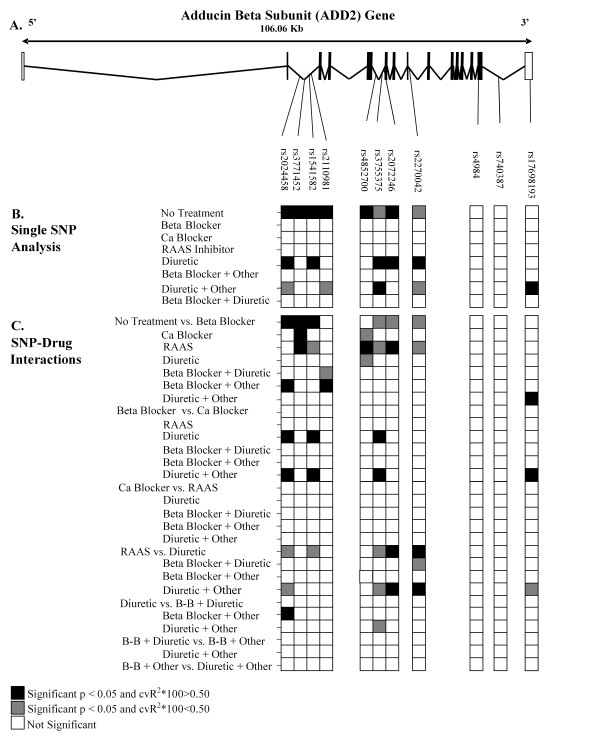
**Distribution of *ADD2 *SNPs on Systolic Blood Pressure within treatment groups and effects of SNP-drug interactions **A: The location of the 11 SNPs on the *ADD2 *gene. B: Results from the analysis of each SNP on systolic blood pressure within each drug class. C: Results of the tests for interaction for each pair of drug classes.

SNP genotyping for subjects from Rochester, MN was conducted at the GENOA central genotyping center at the University of Texas-Houston. SNPs were selected in positional candidate genes in the region of chromosome 2 using the public NCBI database [19] and the private Celera database [20]. SNP genotyping on a total of eleven loci in *ADD2 *gene (Figure 1A) was obtained using a combination of two genotyping platforms: mass spectrometer-based detection system implemented on a Sequenom MassARRAY system, and the fluorogenic TaqMan assay implemented on an ABI Prism 7900 Sequence Detection System. Primer and probe sequences are available from the authors upon request.

### Statistical analysis

The goal of this analysis was to determine if there was evidence of significant SNP and drug main effects and gene-by-drug interactions on systolic BP in hypertensives. Because of the potentially correlated nature of the data (given that subjects were sampled in sibships), we first assessed the distribution of related subjects within each of the ten treatment groups (no treatment, five monotherapy groups and four combination therapy groups). We found that there were few related individuals within the treatment groups (Table 1). It is known that the parameter estimates in regression modeling are not affected by correlated data, however the standard errors of parameters are underestimated [21]. We next examined differences in the age, BMI, systolic and diastolic BP distributions across treatment groups using an *F*-test. Finally, to determine if two SNPs were likely to be representing the same variation in the gene, we calculated r2=D2p1p2q1q23
 MathType@MTEF@5@5@+=feaafiart1ev1aqatCvAUfKttLearuWrP9MDH5MBPbIqV92AaeXatLxBI9gBaebbnrfifHhDYfgasaacH8akY=wiFfYdH8Gipec8Eeeu0xXdbba9frFj0=OqFfea0dXdd9vqai=hGuQ8kuc9pgc9s8qqaq=dirpe0xb9q8qiLsFr0=vr0=vr0dc8meaabaqaciaacaGaaeqabaqabeGadaaakeaacqWGYbGCdaahaaWcbeqaaiabikdaYaaakiabg2da9maalaaabaGaemiraq0aaWbaaSqabeaacqaIYaGmaaaakeaacqWGWbaCdaWgaaWcbaGaeGymaedabeaakiabdchaWnaaBaaaleaacqaIYaGmaeqaaOGaemyCae3aaSbaaSqaaiabigdaXaqabaGccqWGXbqCdaWgaaWcbaGaeGOmaidabeaaaaGcdaahaaWcbeqaaiabiodaZaaaaaa@3DF8@ as a measure of linkage disequilibrium (LD) between SNPs. SNPs with high *r*^2 ^values are likely to have the same genotype-phenotype relationship.

**Table 1:** Distribution of antihypertensive therapies

**Antihypertensive class**	**N**	**Number of sibships**	**Age (yrs)**	**BMI (kg/m**^2^**)**	**Systolic Blood Pressure (mmHg)**	**Diastolic Blood Pressure (mmHg)**
			**Mean (SD)**	**Mean (SD)**	**Mean (SD)**	**Mean (SD)**
	14		54.45	30.9		
No Treatment	8	133	(11.19)	(5.6)	149.45 (12.98)	86.47 (9.40)
Mono Therapies						
	16		55.28	30.7		
Beta-Blocker	2	147	(10.38)	(6.7)	134.77 (16.03)	79.00 (8.13)
			56.61	29.5		
CA-Blocker	62	59	(9.34)	(5.3)	143.08 (15.34)	82.31 (8.99)
	12		57.48	32.0		
Diuretic	8	122	(9.56)	(6.6)	135.27 (12.27)	79.20 (9.38)
	15		54.32	31.1		
RAAS Inhibitors	1	136	(9.52)	(7.3)	134.13 (15.95)	80.64 (8.87)
Combination Therapies						
	16		59.22	32.0		
Beta-Blocker + Diuretic	7	141	(9.47)	(6.8)	136.81 (16.25)	77.55 (9.51)
			61.67	30.7		
Beta-Blocker + Other	73	68	(9.11)	(5.9)	143.43 (20.40)	78.41 (9.37)
	18		58.42	31.7		
Diuretic + Other	0	160	(9.61)	(6.4)	135.06 (15.60)	78.04 (10.01)
Neither Beta-Blocker	44	42	57.70	30.9	138.81 (18.95)	80.45 (10.55)
Nor Diuretic			(9.35)	(6.1)		
			59.61	29.4		
Other Antihypertensive	18	17	(10.62)	(4.8)	141.93 (23.01)	78.52 (8.81)
P-value test among drug classes*			<.001	0.15	<.001	0.01

* ANOVA did not include the "no treatment" class

We next tested for SNP effects on mean levels of systolic BP within each treatment group using linear regression modeling, where SNP genotypes were dummy coded. Because of the limited sample size of some of the drug categories, we used a leave-one-out cross-validation strategy to provide more accurate estimates of the percentage of variation in systolic BP explained by the SNPs and to reduce reporting of false positives. The leave-one-out cross-validation procedure leaves one person out of the sample (designated as the test case) and estimates the genetic model based on the N-1 remaining individuals (designated as the training cases). The estimated model was then applied to the test case, a predicted systolic BP was calculated, and the residual variability between observed and predicted values was estimated. A cross-validated R^2 ^value was then calculated by taking the total systolic BP variation in the sample minus the total residual variation divided by the total variation. The cross-validated R^2 ^may have a negative value when the model's prediction is poor (i.e. the predicted values deviate substantially from the observed values). We used an R^2 ^× 100 > 0.50 cut point to identify SNPs with potential predictive capabilities based on their performance in the test cases. When the same SNP had a significant effect in multiple drug classes, we considered this finding a replicated effect.

After estimating effects within each drug class, we focused on testing for SNP-drug interactions (i.e. whether the effects of SNPs on systolic BP differ between hypertensives on particular classes of antihypertensive therapy). Using analysis of covariance (ANCOVA) methods, we first tested for overall gene-by-drug interactions using the partial *F*-test, then we tested whether the SNPs effects differed among each drug class using pairwise comparisons.

In order to minimize false inferences, we used a four-fold cross-validation strategy in test cases to estimate the extent to which gene-drug interactions improve prediction of systolic BP levels beyond the influences of the main effects of SNP variation on BP levels. For a particular pair of drugs (e.g. beta-blocker vs. diuretic) the four-fold cross-validation strategy begins by randomly dividing the individuals in a drug class into four equal size groups. Three of the four subgroups from each drug class are combined into a "training" dataset and the ANCOVA modeling strategy outlined above was carried out in this training set to estimate the model parameters that were then applied to the one fourth of the data held out as the "testing" dataset. The percent of variation in BP explained in the testing dataset by applying the training model provides a measure of the predictive ability of the model in an independent test sample. Since there are four ways to choose three of the four groups to create independent training-testing sets, we have four estimates of the percent variation predicted by each SNP and its gene-drug interactions. We took the average of the four as an overall measure of the predictive ability of the effects of these factors.

The leave-one-out cross-validation method is an alternative to the four-fold cross-validation method when there is not a large enough sample size to accurately model the genetic effects in the training set (e.g. see Mushiroda et al. 2005 [22]). When we tested whether the *ADD2 *polymorphism had an effect on blood pressure levels within a treatment class (N's ranged from 62 to 180), we thought the sample sizes too small to carry out the four-fold cross-validation. The general question addressed by these two cross-validation methods is the same – that is, can an independent test case's blood pressure level be predicted by the training model? In the leave-one-out method, the predictive accuracy is tallied across all independent test cases to give a statistical average of the predictive accuracy.

Because the cross-validation method provides an alternative to the adjustment of p-values (which is often conservative and can lead to type II errors), we did not formally adjust p-values for multiple testing. This issue is particularly important in susceptibility gene research since the small effects of relatively common alleles are likely to have the greatest public health impact but are unlikely to achieve p-values that withstand conservative adjustments. By testing the predictive capability of the model on independent test cases, cross-validation provides a more direct assessment of whether the result is a false positive.

## Results

Figure 1A presents the location of the eleven SNPs that were genotyped in this study. One synonymous SNP is located in exon 15, nine SNPs are located in introns and one SNP is located in the 3' untranslated region. The average age, BMI, systolic BP and diastolic BP for individuals in each drug class are presented in Table 1, along with a corresponding analysis of variance to determine if the sample means of these traits differ significantly among drug classes. Mean age differed significantly (*p *< 0.001) among the drug classes with "beta-blocker & other" having the oldest individuals on average and RAAS inhibitors the youngest. Overall, there was a significant effect (*p *< 0.001) of drug class on systolic BP, with "beta-blocker + other" associated with the highest mean levels and RAAS inhibitors associated with the lowest mean levels of systolic BP. Table 2 presents the total number of individuals who were typed for each SNP and the frequency and percentage of each of the three genotypes. The numbers of individuals in the monotherapy class of "other" antihypertensive drugs (N = 18) and the combination therapy class "neither beta-blocker nor diuretic" (N = 44) were too small for genetic analyses and therefore were excluded from the test of analyses.

**Table 2:** SNP sample size information

**SNP**	**N**_total_	**N**_11 _**(P**_11_**)**	**N**_12 _**(P**_12_**)**	**N**_22_**(P**_22_**)**
rs2024458	983	68 (0.07)	382 (0.39)	533 (0.54)
rs3771452	936	414 (0.44)	410 (0.44)	112 (0.12)
rs1541582	945	531 (0.56)	350 (0.37)	64 (0.07)
rs2110981	1020	506 (0.50)	418 (0.41)	96 (0.09)
rs4852700	1000	611 (0.61)	353 (0.35)	36 (0.04)
rs3755375	936	514 (0.55)	364 (0.39)	58 (0.06)
rs2072246	981	549 (0.56)	374 (0.38)	58 (0.06)
rs2270042	988	544 (0.55)	384 (0.39)	60 (0.06)
rs4984	977	12 (0.01)	187 (0.19)	778 (0.80)
rs740387	977	777 (0.80)	188 (0.19)	12 (0.01)
rs17698193	997	681 (0.68)	285 (0.29)	31 (0.03)

N: number of subjects

P: genotype frequency

Figure 1B presents the results from the analysis of each SNP on systolic BP within a drug class as well as the results from the leave-one-out cross-validation procedure. Interestingly, there was replicate evidence of cross-validated SNP effects on systolic BP in three of the eight drug categories. In the "no treatment" subgroup of hypertensives, eight of the eleven SNPs appear to be significantly associated with systolic BP levels and six of these SNPs predicted between 2.45% and 4.04% of the variation in systolic BP in the test cases. Some of these same SNPs also predict levels of systolic BP in the treatment groups. Among hypertensives taking a diuretic, there were five SNPs that were significantly associated with systolic BP and all of them predicted a substantial amount of variation in systolic BP in test cases, (cross-validated R^2 ^ranged between 2.66% and 6.27%). In the hypertensives taking "diuretics + other antihypertensive", five SNPs were significantly associated with systolic BP, but only two of them cross-validated (cross-validated R^2 ^was 1.88% and 1.94% for the SNPs rs17698193 and rs3755375, respectively). The only SNP that showed cross-validated evidence of predicting systolic BP levels in all three groups was rs3755375, though in the "no treatment" group it fell significantly below our cut point with a cross-validated R^2 ^× 100 = 0.28. These findings alone provide preliminary evidence for gene-by-drug interactions between *ADD2 *and antihypertensive agents in determining systolic BP levels. A complete description of the systolic BP mean levels associated with each SNP genotype is available online as a data-supplement [see Additional file 1].

In order to formally evaluate whether there was evidence of SNP-by-drug interactions, we compared the effect of each SNP on systolic BP between each drug class and used a four-fold cross-validation strategy to reduce false positives. In Figure 1C, we summarize the results of the tests for interaction for each pair of drug classes examined. Some of the strongest evidence for gene-by-drug interaction comes from the comparison of SNP genotype means in the "no treatment" class relative to the antihypertensive drug classes. This is not surprising since many SNPs have an effect in the "no treatment" class, but do not seem to have an influence in most of the drug classes. This is especially evident when comparing "no treatment" to the beta-blocker treatment group. In particular, the SNP rs2024458 genotypes are associated with a different profile of mean systolic BP levels in the "beta-blocker monotherapy" group and the "beta-blocker + other antihypertensive" group relative to the "no treatment" group. Also, SNP rs3771452 appears to have different mean systolic BP profiles in multiple drug classes, namely, beta-blockers, calcium channel blockers, and RAAS inhibitors, compared to the "no treatment" group. This finding provides initial evidence that gene effects on BP in untreated hypertensives may be very different from gene effects in treated hypertensives. There was also SNP-by-drug interaction when comparing the SNP genotype means in the "beta-blocker monotherapy" group compared to the "diuretic monotherapy" group and "diuretic + other" antihypertensive agent.

**Figure 2:**
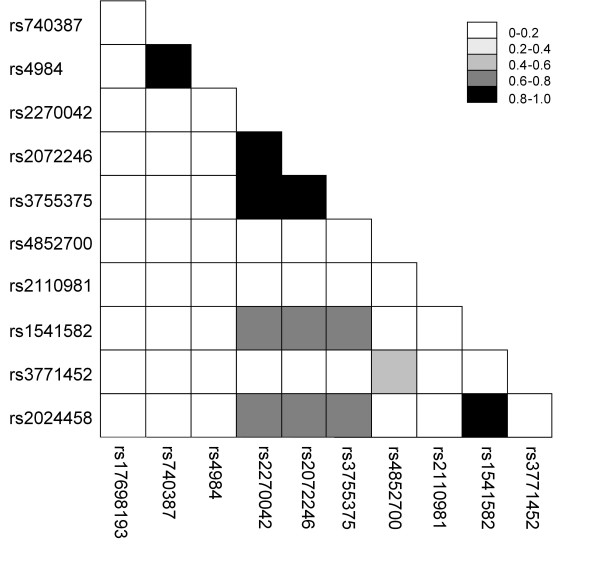
Pattern of linkage disequilibrium (r2) between the 11 SNPs in the *ADD2 *gene.

The linkage disequilibrium pattern among variations in the *ADD2 *SNPs is displayed in Figure 2. It is evident that the relative frequency of several of the SNPs are significantly correlated and thus the effects of each SNP alone is likely to represent influences from multiple SNPs in this region, either measured or unmeasured in this study. There are three groups of SNPs that can be identified by the linkage disequilibrium estimates (*r*^2 ^> 0.95) that are likely to be measuring the same functional variation: group 1 (rs2024458 and rs1541582), group 2 (rs3755375, rs2072246, and rs2270042) and group 3 (rs740387 and rs4984).

**Figure 3:**
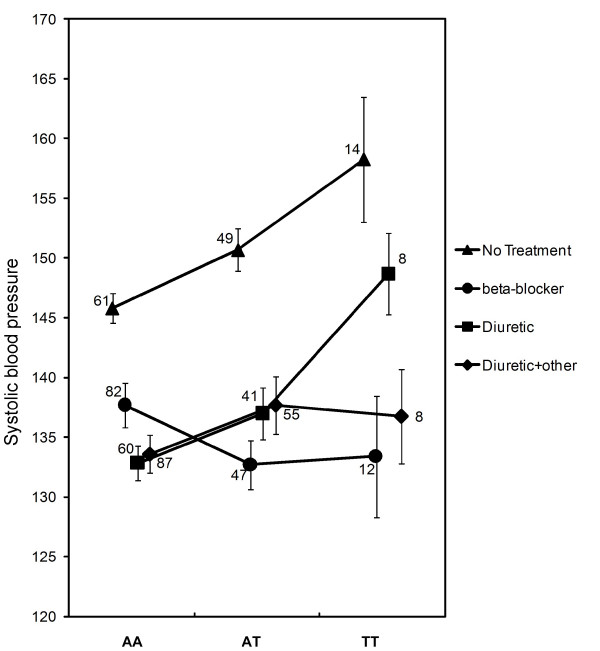
**rs1541582 SNP by drug interactions influencing systolic blood pressure**. Mean levels of systolic blood pressure (mmHg) for the three SNP genotypes (AA, AT and TT) are shown for the "no treatment" (▲), "diuretic" (■), "diuretic + other antihypertensive" (◆) and "beta-blocker" (●) treatment groups. The error bars indicate the standard error of each drug-genotype class. The numbers indicate the number of subjects within each drug-genotype class.

Overall, there were 38 out of 308 tests (11 SNPs*28 pairwise comparisons) that showed significant interactions between genetic variations in the *ADD2 *gene and drug class at a α ≤ 0.05 level of significance. We expected 15 of these significant interactions to have arisen by chance alone (308*0.05 = 15.4). Using the cross-validation approach, we found that 16 of the 38 significant test results did not cross-validate which is consistent with the expectation that 15 of the tests arise by chance alone. Thus, the 22 cases that had cross-validated evidence of gene-by-drug interactions are more likely to represent true positive results. In particular, three SNPs (rs2024458, rs3755375, and rs1541582) have consistent cross-validated differences between beta-blockers and "diuretic monotherapy" and "diuretics + other" antihypertensive combination regimes. These results suggest the differences in mean BP levels depend on both a person's genotype and upon the drug that is administered (Figure 3). In Figure 3 we illustrate this point for SNP rs1541582. We found replicable evidence that the AT and TT genotype classes are associated with higher systolic BP in three drug classes – "no treatment", "diuretics" and "diuretic + others". However, people with the AT or TT genotypes have lower systolic BP levels in the beta-blocker group compared to these other therapies.

## Discussion

Hypertension is directly associated with morbidity and mortality from stroke, myocardial infarction, and renal failure [23-26]. Despite knowledge of the complications of hypertension and well-recognized benefits of interventions that lower BP [27,28], national surveys document suboptimal rates of hypertension awareness, treatment, and control [1]. One gap in our current knowledge about predictors of BP control comes from the lack of large studies of the general population of hypertensives. While clinical trials are a gold standard for directly assessing BP responsiveness to therapies, they are typically not generalizable to the general population of hypertensives because of restrictive inclusion criteria, short duration of the studies, and restriction to a small set of investigative drugs [29,30].

Although it has been suspected for over 50 years that genetic variation plays an important role in determining interindividual differences in therapeutic and toxic responses to many medications [31], success in identifying particular genes and allelic variants responsible for such differences has been limited [32]. For antihypertensive medications, only a few single gene polymorphisms that have large effects on drug metabolizing enzymes and thereby on BP responses have been identified. Their clinical relevance is diminished, however, because most of the agents whose metabolism is largely affected are no longer used for treatment of hypertension. Instead, for contemporary antihypertensive drugs, most interindividual variation in BP response appears to arise from differences in the targeted biochemical, physiological, and anatomical systems regulating BP rather than in mechanisms of drug disposition. Consequently, there is compelling rationale to elucidate the genetic and environmental determinants of interindividual differences in antihypertensive drug responses – both to aid in the selection of more effective antihypertensive drug therapy and to diagnose the pathogenic mechanisms of hypertension in individual patients [33].

The heterogeneous nature of hypertension is reflected in the high degree of interindividual variability in responses to therapy [34,35]. Biological and positional candidate genes, like the angiotensinogen (*AGT*) gene, are more likely to be associated with differences in the pathophysiology of BP, differential risk of developing hypertension, and variability in BP response to antihypertensives in the population at large. For example, studies already indicate the impact of *AGT *and *ACE *genetic variations on BP responsiveness to *ACE *inhibitors [36,37]. In addition, polymorphisms in the *ADD1 *gene, including the Gly460Trp polymorphism, and polymorphisms in the *GNAS *gene encoding the α subunits of the G proteins have been associated with differences in response to BP medications [38-40].

We suspected that *ADD2 *could be associated with differences in response to different antihypertensives because adducins have been proposed to regulate renal tubular transport of Na^+ ^reabsorption and the development of hypertension [6,7]. Mutations in the adducin α subunit in both humans and rats have been associated with hypertension [41]. Fifty percent of the BP difference between Milan normotensive and hypertensive rat strains has been shown to be due to point mutations in the *ADD1 *and *ADD2 *genes [6]. Some studies have found an association with C1797T polymorphism (rs4984) with hypertension (particularly in the presence of *ADD1 *460Trp allele) and systolic BP, depending upon the gender and genetic background of the subjects being studied [42,43].

One of the short-comings of genetic association studies is that they have often failed to replicate and Manly [44] suggests that internal validation common to good experimental practices is one way to avoid the publication of false positives. In our study, we used cross-validation methods to significantly reduce the chance of false positives. Cross-validation methods were developed in the late 1970's as a way to incorporate a measure of predictive accuracy (and correspondingly, a measure of prediction error) for an estimated model based on its performance predicting the outcome for independent test cases [45]. During the last decade, cross-validation methods have been used widely for everything from robust variable selection in gene expression array studies [46] to reducing false positives in gene-gene interaction studies [47,48] to evaluating the predictive accuracy of molecular or genetic classifiers of disease before clinical implementation [49]. It has become a standard in the field of metabolomic [50], proteomic [51,52], and transcriptomic [14,53] studies because of its ease of execution and its emphasis on prediction in independent test cases as a method of discriminating between true associations from false associations.

We found and cross-validated a new set of *ADD2 *SNPs that may also be involved in interindividual variation in BP control in the general population of hypertensives. One SNP (rs4984) had previously been found to be associated with hypertension and systolic BP in predominantly normotensive general populations [42,43]. However, it was not found to be associated with BP levels in the hypertensive population [54] which is consistent with our findings. In this study, rs4984 was not associated with systolic BP in the "no-treatment" group nor any of the drug classes. And there was no evidence of any gene-by-drug interaction for this SNP. Three SNPs (rs2024458, rs3755375, and rs1541582) showed differences in genotypic effects on systolic BP in beta-blocker users versus diuretic users. Two other SNPs (rs2072246 and rs2270042) also predicted differences in genotypic effects in RAAS users versus the diuretics users. Unfortunately, we did not have the sample size to estimate haplotype effect given the large number of variations and moderate number of individuals in each of the drug classes. Recent work [55] has been done in erythrocytes documenting the role of *ADD2 *on intracellular sodium, potassium, and calcium balance which may be a potential mechanism through which *ADD2 *genotypes exert a differential effect in diuretic users vesus other antihypertensive therapies. For example, the thiazide diuretics inhibit Na^+ ^and Cl^- ^transport in the cortical thick ascending limb and early distal tubule. Thiazides can induce a volume contraction, which leads to enhanced proximal tubule reabsorption of fluid and solutes. Use of thiazides results in an increased absorption of Ca^2+ ^and uric acid by the proximal tubule, ultimately leading to reduced excretion of Ca^2+ ^and uric acid. While there may be some connection in underlying mechanisms determining blood pressure, we caution that this is currently speculation and that more work needs to be done to better understand the mechanisms of *ADD2 *genotypic variation action and interaction with antihypertensive therapies.

In addition to the genetic factors, the environmental factors such as salt intake may contribute to the different associations between populations. In a study to evaluate the influence of *ADD2 *rs4984 SNP on BP in three European populations [42], the two populations with higher sodium excretion showed the association between rs4984 SNP and higher BP. Other environmental factors such as physical activity may also need to be considered to further understand the variation in systolic BP in hypertensives.

We acknowledge that there is a fundamental limitation inherent in our community-based study design since there is no prospective data to document genetic influence on blood pressure response within a person or genotype class. Furthermore, the reason a particular patient is on a particular drug regime is unknowable and could likely just represent the particular prescribing preference of the physician. Because we do not expect genotype to affect prescribing patterns and found similar genotype frequencies across drug groups (analysis not shown), we considered the gene effects we observed within a drug class a reflection of how a particular genotype reacts to a particular drug environment. Although it is possible that these study design issues may bias or lead to false positive findings in our study, we have used cross-validation methods to more accurately separate true and false positives. It seems unlikely that the gene-drug interactions identified in our study as being both statistically significant and accurately predicting blood pressure in independent test cases would result from biased false positive results. Nonetheless, carefully designed prospective studies, where individuals are sampled based on genotype, are an essential next step to externally validating our findings. We also acknowledge that given the extensive variation in the *ADD2 *gene, our investigation of 11 SNPs that were implicated in the Barkley et al. [3] work represents only a limited examination of the contribution of this gene to variation in blood pressure and potential interactions with antihypertensive therapies. However, our results suggest that more extensive study of this gene is warranted to better understand the functional genetic polymorphisms that could be operating within this gene region.

## Conclusion

Our results suggest that *ADD2 *variation may influence BP responses to specific antihypertensive drug therapies and measurement of genetic variation may assist in identifying subgroups of hypertensive patients who will benefit most from particular antihypertensive drug therapies. Given that BP levels are mediated by a network of multiple factors (physiological, biochemical, genetic and environmental), interactions among these factors are likely to play a major role in determining an individual's BP or response to antihypertensive therapies. Further studies in larger clinically representative cohorts are needed to investigate these higher order interactions. However, results of this study indicate that it is now possible to identify genetic variations that play a role in one key environmental interaction, namely BP response to antihypertensive therapies.

## Competing interests

The author(s) declare that they have no competing interests.

## Authors' contributions

SLRK participated in the design and coordination of the study, performed the statistical analysis and drafted the manuscript. YVS participated in the design of the study and drafted the manuscript. SCH, RB, EB and STT participated in the design of the study and helped to draft the manuscript. All authors read and approved the final manuscript.

## Pre-publication history

The pre-publication history for this paper can be accessed here:



## Supplementary Material

Additional file 1 Interactions between the Adducin 2 gene and antihypertensive drug therapies in determining blood pressure in people with hypertension. The tables provided summarize the statistical analysis of the ADD2 SNP effects within antihypertensive treatment group and ADD2 SNP × Drug interactions. Click here for file

## References

[B1] Hajjar I, Kotchen TA (2003). Trends in prevalence, awareness, treatment, and control of hypertension in the united states, 1988–2000. JAMA.

[B2] Chobanian AV, Bakris GL, Black HR, Cushman WC, Green LA, Izzo JL, Jones DW, Materson BJ, Oparil S, Wright JT, Roccella EJ, National Heart Lung Blood Institute Joint National Committee on Prevention, Detection, Evaluation, and Treatment of High Blood Pressure, National High Blood Pressure Education Program Coordinating Committee (2003). The seventh report of the joint national committee on prevention, detection, evaluation, and treatment of high blood pressure: The JNC 7 report. JAMA.

[B3] Barkley RA, Chakravarti A, Cooper RS, Ellison RC, Hunt SC, Province MA, Turner ST, Weder AB, Boerwinkle E, Family Blood Pressure Program (2004). Positional identification of hypertension susceptibility genes on chromosome 2. Hypertension.

[B4] Gilligan DM, Lozovatsky L, Silberfein A (1997). Organization of the human beta-adducin gene (ADD2). Genomics.

[B5] Matsuoka Y, Hughes CA, Bennett V (1996). Adducin regulation. definition of the calmodulin-binding domain and sites of phosphorylation by protein kinases A and C. J Biol Chem.

[B6] Bianchi G, Tripodi G, Casari G, Salardi S, Barber BR, Garcia R, Leoni P, Torielli L, Cusi D, Ferrandi M (1994). Two point mutations within the adducin genes are involved in blood pressure variation. Proc Natl Acad Sci USA.

[B7] Hughes CA, Bennett V (1995). Adducin: A physical model with implications for function in assembly of spectrin-actin complexes. J Biol Chem.

[B8] Hunter DJ (2005). Gene-environment interactions in human diseases. Nat Rev Genet.

[B9] Kardia SL, Bielak LF, Lange LA, Cheverud JM, Boerwinkle E, Turner ST, Sheedy PF, Peyser PA (2006). Epistatic effects between two genes in the renin-angiotensin system and systolic blood pressure and coronary artery calcification. Med Sci Monit.

[B10] Ge D, Zhu H, Huang Y, Treiber FA, Harshfield GA, Snieder H, Dong Y (2007). Multilocus analyses of renin-angiotensin-aldosterone system gene variants on blood pressure at rest and during behavioral stress in young normotensive subjects. Hypertension.

[B11] Sing CF, Stengard JH, Kardia SL (2004). Dynamic relationships between the genome and exposures to environments as causes of common human diseases. World Rev Nutr Diet.

[B12] Mosier CI (1951). Problems and designs of cross-validation. Educational and Psychological Measurement.

[B13] Browne MW (2000). Cross-validation methods. J Math Psychol.

[B14] Feng Z, Prentice R, Srivastava S (2004). Research issues and strategies for genomic and proteomic biomarker discovery and validation: A statistical perspective. Pharmacogenomics.

[B15] FBPP Investigators (2002). Multi-center genetic study of hypertension: The family blood pressure program (FBPP). Hypertension.

[B16] FBPP database. http://www.biostat.wustl.edu/fbpp/FBPP.shtml.

[B17] Melton LJ (1996). History of the rochester epidemiology project. Mayo Clin Proc.

[B18] Medi-Span (1996). Proprietary Concepts Master Drug Data Base Documentation Manual.

[B19] NCBI database. http://www.ncbi.nlm.nih.gov.

[B20] Celera database. http://www.celeradiscoverysystem.com.

[B21] Liang KY, Zeger SL (1986). Longitudinal data analysis using generalized estimating equations. Biometrika.

[B22] Mushiroda T, Saito S, Tanaka Y, Takasaki J, Kamatani N, Beck Y, Tahara H, Nakamura Y, Ohnishi Y (2005). A model of prediction system for adverse cardiovascular reactions by calcineurin inhibitors among patients with renal transplants using gene-based single-nucleotide polymorphisms. J Hum Genet.

[B23] Kannel WB, Belanger AJ (1991). Epidemiology of heart failure. Am Heart J.

[B24] Klag MJ, Whelton PK, Randall BL, Neaton JD, Brancati FL, Ford CE, Shulman NB, Stamler J (1996). Blood pressure and end-stage renal disease in men. N Engl J Med.

[B25] MacMahon S, Peto R, Cutler J, Collins R, Sorlie P, Neaton J, Abbott R, Godwin J, Dyer A, Stamler J (1990). Blood pressure, stroke, and coronary heart disease. part 1, prolonged differences in blood pressure: Prospective observational studies corrected for the regression dilution bias. Lancet.

[B26] Stamler J, Stamler R, Neaton JD (1993). Blood pressure, systolic and diastolic, and cardiovascular risks. US population data. Arch Intern Med.

[B27] Collins R, Peto R, MacMahon S, Hebert P, Fiebach NH, Eberlein KA, Godwin J, Qizilbash N, Taylor JO, Hennekens CH (1990). Blood pressure, stroke, and coronary heart disease. part 2, short-term reductions in blood pressure: Overview of randomised drug trials in their epidemiological context. Lancet.

[B28] Hebert PR, Moser M, Mayer J, Glynn RJ, Hennekens CH (1993). Recent evidence on drug therapy of mild to moderate hypertension and decreased risk of coronary heart disease. Arch Intern Med.

[B29] Williams B (2006). Evolution of hypertensive disease: A revolution in guidelines. Lancet.

[B30] Psaty BM, Weiss NS, Furberg CD (2006). Recent trials in hypertension: Compelling science or commercial speech?. JAMA.

[B31] MOTULSKY AG (1957). Drug reactions enzymes, and biochemical genetics. J Am Med Assoc.

[B32] Weber WW (1997). Pharmacogenetics.

[B33] Laragh JH, Lamport B, Sealey J, Alderman MH (1988). Diagnosis ex juvantibus. individual response patterns to drugs reveal hypertension mechanisms and simplify treatment. Hypertension.

[B34] Turner ST, Schwartz GL, Chapman AB, Boerwinkle E (2001). C825T polymorphism of the G protein beta(3)-subunit and antihypertensive response to a thiazide diuretic. Hypertension.

[B35] Turner ST, Boerwinkle E (2003). Genetics of blood pressure, hypertensive complications, and antihypertensive drug responses. Pharmacogenomics.

[B36] Hingorani AD, Jia H, Stevens PA, Hopper R, Dickerson JE, Brown MJ (1995). Renin-angiotensin system gene polymorphisms influence blood pressure and the response to angiotensin converting enzyme inhibition. J Hypertens.

[B37] Sasaki M, Oki T, Iuchi A, Tabata T, Yamada H, Manabe K, Fukuda K, Abe M, Ito S (1996). Relationship between the angiotensin converting enzyme gene polymorphism and the effects of enalapril on left ventricular hypertrophy and impaired diastolic filling in essential hypertension: M-mode and pulsed doppler echocardiographic studies. J Hypertens.

[B38] Cusi D, Barlassina C, Azzani T, Casari G, Citterio L, Devoto M, Glorioso N, Lanzani C, Manunta P, Righetti M, Rivera R, Stella P, Troffa C, Zagato L, Bianchi G (1997). Polymorphisms of alpha-adducin and salt sensitivity in patients with essential hypertension. Lancet.

[B39] Turner ST, Chapman AB, Schwartz GL, Boerwinkle E (2003). Effects of endothelial nitric oxide synthase, alpha-adducin, and other candidate gene polymorphisms on blood pressure response to hydrochlorothiazide. Am J Hypertens.

[B40] Jia H, Hingorani AD, Sharma P, Hopper R, Dickerson C, Trutwein D, Lloyd DD, Brown MJ (1999). Association of the G(s)alpha gene with essential hypertension and response to beta-blockade. Hypertension.

[B41] Ferrandi M, Tripodi G, Salardi S, Florio M, Modica R, Barassi P, Parenti P, Shainskaya A, Karlish S, Bianchi G, Ferrari P (1996). Renal na, K-ATPase in genetic hypertension. Hypertension.

[B42] Tikhonoff V, Kuznetsova T, Stolarz K, Bianchi G, Casiglia E, Kawecka-Jaszcz K, Nikitin Y, Tizzone L, Wang JG, Staessen JA (2003). Beta-adducin polymorphisms, blood pressure, and sodium excretion in three european populations. Am J Hypertens.

[B43] Wang JG, Staessen JA, Barlassina C, Fagard R, Kuznetsova T, Struijker-Boudier HA, Zagato L, Citterio L, Messaggio E, Bianchi G (2002). Association between hypertension and variation in the alpha- and beta-adducin genes in a white population. Kidney Int.

[B44] Manly KF (2005). Reliability of statistical associations between genes and disease. Immunogenetics.

[B45] Stone M (1974). Cross-validatory choice and assessment of statistical predictions. Journal of the Royal Statistical Society Series B (Methodological).

[B46] Zhu J, Hastie T (2004). Classification of gene microarrays by penalized logistic regression. Biostatistics.

[B47] Ritchie MD, Motsinger AA (2005). Multifactor dimensionality reduction for detecting gene-gene and gene-environment interactions in pharmacogenomics studies. Pharmacogenomics.

[B48] Gong R, Liu Z, Li L (2007). Epistatic effect of plasminogen activator inhibitor 1 and beta-fibrinogen genes on risk of glomerular microthrombosis in lupus nephritis: Interaction with environmental/clinical factors. Arthritis Rheum.

[B49] Larsen JE, Pavey SJ, Passmore LH, Bowman RV, Hayward NK, Fong KM (2007). Gene expression signature predicts recurrence in lung adenocarcinoma. Clin Cancer Res.

[B50] Pohjanen E, Thysell E, Jonsson P, Eklund C, Silfver A, Carlsson IB, Lundgren K, Moritz T, Svensson MB, Antti H A multivariate screening strategy for investigating metabolic effects of strenuous physical exercise in human serum. J Proteome Res.

[B51] Agranoff D, Fernandez-Reyes D, Papadopoulos MC, Rojas SA, Herbster M, Loosemore A, Tarelli E, Sheldon J, Schwenk A, Pollok R, Rayner CF, Krishna S (2006). Identification of diagnostic markers for tuberculosis by proteomic fingerprinting of serum. Lancet.

[B52] Mertens BJ, De Noo ME, Tollenaar RA, Deelder AM (2006). Mass spectrometry proteomic diagnosis: Enacting the double cross-validatory paradigm. J Comput Biol.

[B53] Wood IA, Visscher PM, Mengersen KL (2007). Classification based upon gene expression data: Bias and precision of error rates. Bioinformatics.

[B54] Lanzani C, Citterio L, Jankaricova M, Sciarrone MT, Barlassina C, Fattori S, Messaggio E, Serio CD, Zagato L, Cusi D, Hamlyn JM, Stella A, Bianchi G, Manunta P (2005). Role of the adducin family genes in human essential hypertension. J Hypertens.

[B55] Richart T, Thijs L, Kuznetsova T, Tikhonoff V, Zagato L, Lijnen P, Fagard R, Wang J, Bianchi G, Staessen JA (2007). Intra-erythrocyte cation concentrations in relation to the C1797T beta-adducin polymorphism in a general population. J Hum Hypertens.

