# Supplementary material for: Interactions between the adducin 2 gene and antihypertensive drug therapies in determining blood pressure in people with hypertension
Source: BMC Med Genet. 2007 Sep 13;8:61. doi: 10.1186/1471-2350-8-61 (PMC2065870; doi:10.1186/1471-2350-8-61)
Supplement: Additional file 1 — Interactions between the Adducin 2 gene and antihypertensive drug therapies in determining blood pressure in people with hypertension. The tables provided summarize the statistical analysis of the ADD2 SNP effects within antihypertensive treatment group and ADD2 SNP × Drug interactions. [file 1471-2350-8-61-S1.pdf]

Additional file 1.

Appendix Table I: Therapy Systolic BPs [Mean (SD)] by SNP genotype

	No Treatment									Beta-Blocker								
	SNP11			SNP12			SNP22			SNP11			SNP12			SNP22		
SNP	Mean	SD	N	Mean	SD	N	Mean	SD	N	Mean	SD	N	Mean	SD	N	Mean	SD	N
ADD2_rs740387	149.70	12.53	104	147.77	12.31	23	144.00		1	134.83	16.25	119	135.27	15.50	21	154.33	9.90	2
ADD2_rs2024458	158.04	18.97	15	150.56	11.28	52	146.31	10.36	61	133.39	17.55	12	131.88	13.98	51	136.69	17.23	79
ADD2_rs17698193	149.53	13.22	91	149.15	13.06	37	146.67	3.03	4	133.94	15.72	92	137.43	17.33	48	130.78	12.60	6
ADD2_rs4984	144.00		1	145.52	12.16	22	150.45	13.39	101	154.33	9.90	2	135.64	15.20	22	134.46	16.23	117
ADD2_rs2072246	145.98	10.97	73	153.71	12.35	46	156.00	21.09	11	135.88	16.11	79	134.52	16.34	58	136.67	18.30	8
ADD2_rs3755375	146.24	10.27	61	152.40	12.32	47	156.00	21.09	11	135.76	16.08	70	133.53	16.85	54	133.82	18.34	11
ADD2_rs4852700	151.69	13.67	89	146.30	10.32	38	134.00	8.19	3	135.92	17.29	82	134.54	14.68	58	138.93	14.72	5
ADD2_rs2110981	147.69	10.45	62	148.77	12.73	57	160.53	17.33	15	133.87	16.03	72	134.15	16.65	57	140.58	15.10	15
ADD2_rs1541582	145.81	9.78	61	150.68	12.54	49	158.24	19.67	14	137.71	16.98	82	132.68	14.19	47	133.39	17.55	12
ADD2_rs3771452	152.02	14.19	67	147.48	10.10	47	140.15	11.69	9	134.86	15.78	58	135.06	17.41	59	138.13	13.24	20
ADD2_rs2270042	146.18	11.41	67	152.24	12.03	49	156.07	22.23	10	136.04	16.05	76	133.97	16.43	58	136.74	17.74	9

Appendix Table I: Therapy Systolic BPs [Mean (SD)] by SNP genotype (Continued)

	CA-Blocker									RAAS Inhibitor								
	SNP11			SNP12			SNP22			SNP11			SNP12			SNP22		
SNP	Mean	SD	N	Mean	SD	N	Mean	SD	N	Mean	SD	N	Mean	SD	N	Mean	SD	N
ADD2_rs740387	143.21	14.59	43	144.10	19.66	13			0	134.64	15.23	96	132.80	23.36	20	138.67	24.51	2
ADD2_rs2024458	150.67		1	142.67	14.68	24	146.11	16.22	29	134.44	12.41	3	132.47	13.58	43	135.41	18.14	72
ADD2_rs17698193	143.98	15.38	41	140.05	14.38	13	140.00		1	134.51	17.38	87	134.12	14.95	30	128.33	12.43	4
ADD2_rs4984			0	145.80	21.53	10	143.86	14.57	42	156.00		1	132.81	23.93	19	134.66	14.81	97
ADD2_rs2072246	142.02	16.56	29	145.57	15.20	25	141.67	12.73	2	135.96	18.63	69	132.54	12.59	46	126.67	14.51	5
ADD2_rs3755375	144.40	15.79	27	145.18	15.41	22	141.67	12.73	2	135.30	18.66	64	133.04	13.86	46	129.56	14.24	3
ADD2_rs4852700	141.33	14.35	31	146.64	15.09	23	139.33	26.40	2	132.82	17.14	65	134.73	15.03	53	139.00	21.43	4
ADD2_rs2110981	142.58	15.46	24	142.41	15.67	26	151.60	16.48	5	135.03	17.64	67	133.61	15.38	51	135.83	13.68	8
ADD2_rs1541582	143.72	17.12	31	144.84	14.11	19	145.33	7.54	2	136.43	17.59	69	131.68	14.46	43	134.44	12.41	3
ADD2_rs3771452	139.17	11.48	20	145.05	17.63	19	139.70	12.31	9	134.09	14.59	38	135.72	18.38	61	136.41	13.75	13
ADD2_rs2270042	143.00	15.77	30	146.55	14.60	23			0	136.10	19.18	66	131.24	12.13	48	126.67	14.51	5

Appendix Table I: Therapy Systolic BPs [Mean (SD)] by SNP genotype (Continued)

	Diuretic									Beta-Blocker & Diuretic								
	SNP11			SNP12			SNP22			SNP11			SNP12			SNP22		
SNP	Mean	SD	N	Mean	SD	N	Mean	SD	N	Mean	SD	N	Mean	SD	N	Mean	SD	N
ADD2_rs740387	135.88	12.84	80	132.81	13.72	23	136.33	9.02	4	137.57	16.20	112	136.92	19.13	32	133.33	10.37	2
ADD2_rs2024458	146.81	10.60	9	137.45	13.50	44	132.20	11.00	58	140.73	12.49	10	138.25	17.38	63	136.52	16.98	75
ADD2_rs17698193	134.63	12.29	74	137.12	13.12	34	145.33	10.87	4	136.95	17.48	100	137.65	15.50	47	143.17	6.22	4
ADD2_rs4984	135.20	8.21	5	133.95	12.26	25	136.13	12.89	81	133.33	10.37	2	136.39	17.68	34	136.99	16.23	115
ADD2_rs2072246	132.83	11.36	58	135.91	12.96	47	146.89	10.45	9	136.58	16.88	81	140.26	17.15	54	136.19	12.49	7
ADD2_rs3755375	132.27	11.09	55	136.70	12.92	41	145.58	10.35	8	136.47	17.16	79	140.02	17.26	55	136.19	12.49	7
ADD2_rs4852700	135.09	12.19	74	136.60	12.83	39	133.56	15.98	3	138.14	16.48	88	135.71	16.57	55	136.56	16.04	6
ADD2_rs2110981	135.31	12.48	59	134.94	12.78	53	136.38	9.89	7	138.98	17.20	82	133.65	13.99	54	140.11	19.64	18
ADD2_rs1541582	132.83	11.18	60	136.98	13.73	41	148.67	9.65	8	137.34	16.34	75	139.67	17.31	55	140.73	12.49	10
ADD2_rs3771452	136.11	12.44	41	135.53	12.72	55	133.91	14.16	15	138.32	16.62	65	135.64	16.96	57	135.68	16.47	19
ADD2_rs2270042	132.66	11.19	61	136.54	13.73	43	144.87	11.74	10	136.07	16.72	83	139.52	16.44	57	139.33	14.59	8

Appendix Table I: Therapy Systolic BPs [Mean (SD)] by SNP genotype (Continued)

	Beta-Blocker & Other									Diuretic & Other								
	SNP11			SNP12			SNP22			SNP11			SNP12			SNP22		
SNP	Mean	SD	N	Mean	SD	N	Mean	SD	N	Mean	SD	N	Mean	SD	N	Mean	SD	N
ADD2_rs740387	144.87	19.94	50	139.69	21.60	15			0	134.94	15.65	129	138.29	16.92	30			0
ADD2_rs2024458	134.40	14.95	5	147.14	18.86	28	143.19	21.18	36	136.75	11.28	8	138.60	17.43	59	133.20	15.38	88
ADD2_rs17698193	143.03	20.74	50	147.46	20.65	16	138.00	11.31	2	134.06	15.73	109	138.44	14.07	46	154.22	4.44	3
ADD2_rs4984			0	138.67	22.04	14	143.40	19.29	52			0	137.64	17.03	30	134.71	15.70	129
ADD2_rs2072246	143.33	21.32	33	144.08	18.54	26	146.67	20.78	5	133.97	14.74	93	138.69	17.07	56	140.27	12.42	5
ADD2_rs3755375	142.76	21.47	34	145.31	18.45	30	146.67	20.78	5	132.54	15.76	89	139.65	17.03	54	129.73	3.42	5
ADD2_rs4852700	144.94	21.19	49	141.83	16.41	16	153.00	33.47	2	135.82	15.26	100	135.11	17.49	54	128.00	15.78	6
ADD2_rs2110981	147.04	20.51	36	139.42	21.17	23	143.42	15.09	8	134.46	17.05	79	133.79	13.84	70	144.24	17.13	14
ADD2_rs1541582	142.10	21.23	33	140.21	17.60	25	138.83	12.92	4	133.59	14.95	87	137.68	17.92	55	136.75	11.28	8
ADD2_rs3771452	144.09	20.40	37	143.16	20.01	23	143.33	28.11	5	135.16	15.82	65	134.01	14.81	68	135.02	14.48	15
ADD2_rs2270042	141.52	20.78	33	140.59	17.55	27	146.67	20.78	5	133.00	15.79	92	138.60	16.26	62	140.19	11.67	7

Appendix Table II: SNP effects on Systolic BP within Treatment class (R^2*100)

	No Treatment					Beta-Blocker					CA-Blocker					RAAS Inhibitor				
SNP	R^2	1	2	3	CV R^2	R^2	1	2	3	CV R^2	R^2	1	2	3	CV R^2	R^2	1	2	3	CV R^2
ADD2_rs740387	0.50				-2.84	2.03				-1.54						0.28				-9.96
ADD2_rs2024458	**8.99**	**	*		**2.82**	1.99				-2.42	1.54				-6.57	0.73				-3.76
ADD2_rs17698193	0.15				-3.13	1.30				-2.53	1.34				-6.26	0.44				-3.86
ADD2_rs4984	2.19				-1.04	2.18				-1.40						1.63				-4.98
ADD2_rs2072246	**9.86**	**	*		**3.15**	0.20				-4.49	1.31				-9.87	1.94				-2.70
ADD2_rs3755375	**7.82**	**	*		0.28	0.44				-4.50	0.22				-12.48	0.66				-4.47
ADD2_rs4852700	**6.95**	*	*		**3.50**	0.34				-3.73	3.31				-20.90	0.67				-5.92
ADD2_rs2110981	**9.36**	**	*	*	**4.04**	1.56				-2.57	2.88				-9.34	0.22				-4.24
ADD2_rs1541582	**9.62**	**	*	*	**3.14**	2.23				-2.20	0.14				-9.30	1.94				-2.72
ADD2_rs3771452	**6.86**	*	*		**2.45**	0.49				-3.69	3.89				-8.82	0.27				-4.67
ADD2_rs2270042	**7.13**	*	*		-0.29	0.44				-4.19						2.82				-1.83

1 = partial F-test associated with SNP by drug interaction

2 = t-test associated with interaction represented SNP_{11} by drug interaction

3 = t-test associated with interaction represented SNP_{12} by drug interaction

+ = p-value < 0.1

* = p-value < 0.05

** = p-value < 0.01

Appendix Table II: SNP effects on Systolic BP within Treatment class (R^2*100) (Continued)

SNP	Diuretic					Beta-Blocker & Diuretic					Beta-Blocker & Other					Diuretic & Other				
	R^2	1	2	3	CV R^2	R^2	1	2	3	CV R^2	R^2	1	2	3	CV R^2	R^2	1	2	3	CV R^2
ADD2_rs740387	0.99				-4.17	0.11				-3.82										
ADD2_rs2024458	**11.11**	**	*	*	**6.27**	0.51				-3.13	2.82				-5.11	**2.60**			*	-0.79
ADD2_rs17698193	2.98				-2.31	0.37				-2.49	1.11				-6.53	**4.47**	*	*		**1.94**
ADD2_rs4984	0.53				-4.06	0.08				-3.59										
ADD2_rs2072246	**9.01**	**	*	*	**4.27**	1.16				-2.62	0.20				-10.22	2.34				-1.32
ADD2_rs3755375	**9.20**	**	*		**3.93**	1.06				-2.73	0.51				-9.14	4.72	*			**1.88**
ADD2_rs4852700	0.40				-6.79	0.51				-3.66	0.99				-16.89	0.85				-3.15
ADD2_rs2110981	0.08				-4.65	2.60				-1.65	3.03				-5.34	**3.22**	+	*	*	-0.63
ADD2_rs1541582	**10.89**	**	*	*	**6.12**	0.60				-3.32	0.33				-8.31	1.54				-2.06
ADD2_rs3771452	0.30				-5.63	0.64				-3.71	0.05				-12.71	0.14				-3.96
ADD2_rs2270042	**7.78**	*	*		**2.66**	1.07				-2.84	0.66				-9.79	3.24	+			-0.01

Appendix Table III: SNP-by-genotype interactions on Systolic BP: No treatment vs. Antihypertensive therapies (R^2*100)

SNP	Beta-Blocker					CA-Blocker					RAAS Inhibitor					Diuretic				
	R^2	1	2	3	CV R^2	R^2	1	2	3	CV R^2	R^2	1	2	3	CV R^2	R^2	1	2	3	CV R^2
ADD2_rs740387	0.66				-1.82						0.09				-0.29	0.09				-0.95
ADD2_rs2024458	2.74	**	*	*	**1.00**	1.64				-1.38	1.43				-0.62	0.09				-4.51
ADD2_rs17698193	0.30				-0.16	0.25				-1.83	0.03				-1.82	0.82				-0.53
ADD2_rs4984	1.06				-0.93						0.67				-0.98	0.18				0.08
ADD2_rs2072246	1.90	*			-1.16	0.75				-1.54	**3.56**	**	*		**0.87**	0.87				-4.27
ADD2_rs3755375	1.90	*			-0.54	1.27				-0.20	1.97	+			-1.42	0.25				-5.93
ADD2_rs4852700	1.25				0.20	3.25	*			0.06	**2.13**	*	*		**2.17**	1.74	+			-0.02
ADD2_rs2110981	0.31				-0.89	0.13				-5.45	0.94				0.33	1.08				-0.24
ADD2_rs1541582	3.38	**	*		**1.43**	0.77				-0.23	2.35	*			0.24	0.15				-4.58
ADD2_rs3771452	1.84	+	*		**1.48**	3.11	+			**2.19**	**1.69**	+	*		**1.04**	1.01				-1.89
ADD2_rs2270042	1.60	+			-2.13						**3.42**	**	*		-0.11	0.22				-4.71

1 = partial F-test associated with SNP by drug interaction

2 = t-test associated with interaction represented SNP_{11} by drug interaction

3 = t-test associated with interaction represented SNP_{12} by drug interaction

+ = p-value < 0.1

* = p-value < 0.05

** = p-value < 0.01

Appendix Table III: SNP-by-genotype interactions on Systolic BP: No treatment vs. Antihypertensive therapies (R^2*100) (Continued)

	Beta-Blocker & Diuretic					Beta-Blocker & Other					Diuretic & Other				
SNP	R^2	1	2	3	CV R^2	R^2	1	2	3	CV R^2	R^2	1	2	3	CV R^2
ADD2_rs740387	0.03				-0.38										
ADD2_rs2024458	0.45				-0.37	**2.98**	*	*		**0.54**	0.52				-0.22
ADD2_rs17698193	0.23				-1.25	0.40				-0.96	**1.53**	+	*		**1.52**
ADD2_rs4984	0.28				-0.07										
ADD2_rs2072246	0.79				-0.17	1.06				-3.06	0.22				-1.23
ADD2_rs3755375	0.61				-0.46	0.39				-4.29	0.77				-0.05
ADD2_rs4852700	0.79				-0.19	1.56				-1.87	0.60				0.07
ADD2_rs2110981	**1.62**	+	*		-0.01	**2.93**	*	*		**1.22**	0.11				-1.96
ADD2_rs1541582	0.66				-0.43	1.97				-2.25	0.54				-0.66
ADD2_rs3771452	0.63				-1.06	0.82				-7.83	1.03				0.28
ADD2_rs2270042	0.33				-0.81	1.01				-5.85	0.04				-1.80

Appendix Table IV: SNP-by-genotype interactions on Systolic BP: Antihypertensive therapies vs. Antihypertensive therapies (R^2*100)

	Beta-Blocker vs.CA-Blocker					Beta-Blocker vs. RAAS Inhibitor					Beta-Blocker vs. Diuretic					CA-Blocker vs. RAAS Inhibitor					CA-Blocker vs. Diuretic				
SNP	R^2	1	2	3	CV R^2	R^2	1	2	3	CV R^2	R^2	1	2	3	CV R^2	R^2	1	2	3	CV R^2	R^2	1	2	3	CV R^2
ADD2_rs740387						0.38				-2.08	1.01				-2.28										
ADD2_rs2024458	0.12				-0.72	0.08				-1.00	**4.20**	**	*	*	**3.52**	0.06				-0.24	2.07				-3.39
ADD2_rs17698193	0.79				-1.96	0.29				-1.14	0.86				-0.66	0.19				-3.86	1.32				-1.40
ADD2_rs4984						0.12				-1.12	1.25				-2.11										
ADD2_rs2072246	0.46				-2.12	0.45				-0.33	1.44				-0.56	0.99				-2.56	1.00				-7.58
ADD2_rs3755375	0.17				-2.02	0.05				-1.00	**2.40**	+	*		**1.01**	0.17				-2.90	1.40				-7.16
ADD2_rs4852700	0.97				-5.07	0.25				-1.72	0.32				-2.24	0.47				-4.46	0.40				-8.48
ADD2_rs2110981	0.04				-5.19	0.24				-0.79	0.24				-0.47	0.35				-2.18	0.49				-6.28
ADD2_rs1541582	0.66				0.18	0.02				-0.64	**4.32**	**	*		**4.12**	0.61				-0.06	1.02				-3.18
ADD2_rs3771452	0.85				-0.24	0.07				-0.74	0.37				-1.49	0.47				-0.99	1.01				-2.66
ADD2_rs2270042						0.51				-0.67	1.56				**0.62**										

1 = partial F-test associated with SNP by drug interaction

2 = t-test associated with interaction represented SNP_{11} by drug interaction

3 = t-test associated with interaction represented SNP_{12} by drug interaction

+ = p-value < 0.1

* = p-value < 0.05

** = p-value < 0.01

Appendix Table IV: SNP-by-genotype interactions on Systolic BP: Antihypertensive therapies vs. Antihypertensive therapies (R^2*100) (Continued)

	RAAS Inhibitor vs. Diuretic					Beta-Blocker vs. Beta-Blocker & Diuretic					Beta-Blocker vs. Beta-Blocker & Other Antihypertensive					Beta-Blocker vs. Diuretic & Other Antihypertensive					CA-Blocker vs. Beta-Blocker & Diuretic				
SNP	R^2	1	2	3	CV R^2	R^2	1	2	3	CV R^2	R^2	1	2	3	CV R^2	R^2	1	2	3	CV R^2	R^2	1	2	3	CV R^2
ADD2_rs740387	0.05				-3.55	0.72				-1.21															
ADD2_rs2024458	**2.43**	+		*	-0.68	1.02				-0.43	1.58				-0.50	**2.27**	*		*	**1.89**	0.47				-0.59
ADD2_rs17698193	1.17				-1.80	0.46				-1.68	0.02				-1.72	**1.39**		*		**0.83**	0.41				-1.52
ADD2_rs4984	0.82				-3.36	0.73				-1.02															
ADD2_rs2072246	**3.72**	*	*		**1.91**	0.58				-0.71	0.08				-2.38	0.88				0.38	0.00				-1.43
ADD2_rs3755375	2.38	+			-0.77	0.68				-0.65	0.42				-0.72	1.94	+			**1.47**	0.13				-0.83
ADD2_rs4852700	0.19				-2.79	0.08				-0.92	0.10				-6.14	0.42				-1.61	1.09				-3.84
ADD2_rs2110981	0.03				-1.80	0.73				-1.98	1.23				-0.21	0.12				-0.79	0.60				-3.21
ADD2_rs1541582	2.96	*			-0.24	1.27				0.23	0.16				-1.56	1.86	+			**1.27**	0.03				-0.41
ADD2_rs3771452	0.25				-2.05	0.39				-0.25	0.09				-5.93	0.12				-1.48	1.11				-0.07
ADD2_rs2270042	**3.99**	**	*		**2.73**	0.66				-0.86	0.10				-2.10	1.36				**0.59**					

Appendix Table IV: SNP-by-genotype interactions on Systolic BP: Antihypertensive therapies vs. Antihypertensive therapies (R^2*100) (Continued)

	CA-Blocker vs. Beta-Blocker & Other Antihypertensive					CA-Blocker vs. Diuretic & Other Antihypertensive					RAAS Inhibitor vs. Beta-Blocker & Diuretic					RAAS Inhibitor vs. Beta-Blocker & Other Antihypertensive					RAAS Inhibitor vs. Diuretic & Other Antihypertensive				
SNP	R^2	1	2	3	CV R^2	R^2	1	2	3	CV R^2	R^2	1	2	3	CV R^2	R^2	1	2	3	CV R^2	R^2	1	2	3	CV R^2
ADD2_rs740387											0.11				-0.70										
ADD2_rs2024458	1.56				0.01	1.37				-0.47	0.48				-1.37	1.08				-0.46	**1.48**			*	-0.31
ADD2_rs17698193	0.97				-1.23	1.71				-1.24	0.40				-0.84	0.28				-2.58	**1.91**	+	*		0.23
ADD2_rs4984											0.60				-0.90										
ADD2_rs2072246	0.23				-4.30	0.12				-1.95	1.17				-1.11	0.72				-1.63	1.99	+			**0.96**
ADD2_rs3755375	0.17				-3.18	0.68				-0.49	0.71				-2.67	0.53				-0.41	1.86	+			-0.27
ADD2_rs4852700	1.54				-5.56	0.65				-3.54	0.52				-1.02	0.36				-5.05	0.68				-0.25
ADD2_rs2110981	1.50				-1.38	0.01				-6.74	0.33				-1.36	0.58				-0.51	0.48				-2.41
ADD2_rs1541582	0.21				-1.66	0.14				-0.97	1.06				-0.49	0.16				-2.74	1.69				0.39
ADD2_rs3771452	0.75				-7.58	0.91				-0.55	0.42				-1.15	0.11				-6.88	0.17				-1.92
ADD2_rs2270042											1.77	+			-1.23	0.91				-3.36	2.93	*			**1.91**

Appendix Table IV: SNP-by-genotype interactions on Systolic BP: Antihypertensive therapies vs. Antihypertensive therapies (R^2*100) (Continued)

	Diuretic vs. Beta-Blocker & Diuretic					Diuretic vs. Beta-Blocker & Other Antihypertensive					Diuretic vs. Diuretic & Other Antihypertensive					Beta-Blocker & Diuretic vs. Beta-Blocker & Other Antihypertensive					Beta-Blocker & Diuretic vs. Diuretic & Other Antihypertensive					Beta-Blocker & Other Antihypertensive vs. Diuretic & Other Antihypertensive				
SNP	R^2	1	2	3	CV R^2	R^2	1	2	3	CV R^2	R^2	1	2	3	CV R^2	R^2	1	2	3	CV R^2	R^2	1	2	3	CV R^2	R^2	1	2	3	CV R^2
ADD2_rs740387	0.16				-1.34																									
ADD2_rs2024458	0.88				-2.36	**3.28**	*	*		**0.68**	0.85				-2.61	0.93				-0.31	0.31				-0.68	0.59				-1.43
ADD2_rs17698193	0.12				-2.13	0.77				**1.08**	0.33				-1.41	0.44				-1.21	0.62				-0.57	1.09				0.47
ADD2_rs4984	0.07				-1.16																									
ADD2_rs2072246	1.39				-1.72	0.72				-2.12	0.47				-2.26	0.23				-0.95	0.17				-0.90	0.25				-2.54
ADD2_rs3755375	1.11				-3.00	0.53				-3.02	**1.77**			*	-1.68	0.10				-1.80	0.32				-0.85	0.58				-1.85
ADD2_rs4852700	0.40				-1.55	0.65				-5.52	0.22				-1.85	0.21				-5.62	0.23				-1.55	0.62				-1.74
ADD2_rs2110981	0.65				-1.46	1.03				-0.93	0.58				-0.38	0.17				-1.38	0.82				-2.31	1.38				-0.59
ADD2_rs1541582	1.10				-2.89	2.45				-1.91	1.08				-2.07	0.39				-1.02	0.07				-0.98	0.64				-2.27
ADD2_rs3771452	0.11				-2.37	0.02				-4.96	0.08				-2.84	0.05				-4.63	0.08				-2.25	0.00				-4.31
ADD2_rs2270042	0.55				-2.19	0.67				-4.00	0.29				-1.97	0.37				-1.79	0.14				-0.98	0.68				-3.17
